# HSPA9 reduction exacerbates symptoms and cell death in DSS-Induced inflammatory colitis

**DOI:** 10.1038/s41598-024-56216-w

**Published:** 2024-03-11

**Authors:** Soyoung Jang, Soyeon Jang, Jiwon Ko, Ji-Eun Bae, Hyejin Hyung, Ji Yeong Park, Su-Geun Lim, Sijun Park, Song Park, Junkoo Yi, Seonggon Kim, Myoung Ok Kim, Dong-Hyung Cho, Zae Young Ryoo

**Affiliations:** 1 School of Life Sciences, BK21 FOUR KNU Creative BioResearch Group, Kyungpook National University, Daegu, 41566 Republic of Korea; 2 KNU LAMP Research Center, KNU Institute of Basic Sciences, College of Natural Sciences, Kyungpook National University, Daegu, 41566 Republic of Korea; 3 Institute of Life Science and Biotechnology, Kyungpook National University, Daegu, 41566 Republic of Korea; 4 Department of Animal Science, Gyeongsang National University, Jinju, 52828 Republic of Korea; 5 Institute of Agriculture and Life Science (IALS), Gyeongsang National University, Jinju, 52828, Republic of Korea; 6 School of Animal Life Convergence Science, Hankyong National University, Anseong, 17579 Korea; 7 Preclinical Research Center, Daegu-Gyeongbuk Medical Innovation Foundation, Daegu, Korea; 8 Department of Animal Science and Biotechnology, Research Institute for Innovative Animal Science, Kyungpook National University, Sangju-si, Gyeongsang buk-do 37224, Republic of Korea; 9 Organelle Institute, Kyungpook National University, Daegu, 41566, Republic of Korea; 10 ORGASIS Corp., Suwon, Gyeonggido 16229, Republic of Korea

**Keywords:** Immunology, Gastrointestinal diseases

## Abstract

Inflammatory bowel disease (IBD) is a chronic inflammatory condition that is influenced by various factors, including environmental factors, immune responses, and genetic elements. Among the factors that influence IBD progression, macrophages play a significant role in generating inflammatory mediators, and an increase in the number of activated macrophages contributes to cellular damage, thereby exacerbating the overall inflammatory conditions. HSPA9, a member of the heat shock protein 70 family, plays a crucial role in regulating mitochondrial processes and responding to oxidative stress. HSPA9 deficiency disrupts mitochondrial dynamics, increasing mitochondrial fission and the production of reactive oxygen species. Based on the known functions of HSPA9, we considered the possibility that HSPA9 reduction may contribute to the exacerbation of colitis and investigated its relevance. In a dextran sodium sulfate-induced colitis mouse model, the downregulated HSPA9 exacerbates colitis symptoms, including increased immune cell infiltration, elevated proinflammatory cytokines, decreased tight junctions, and altered macrophage polarization. Moreover, along with the increased mitochondrial fission, we found that the reduction in HSPA9 significantly affected the superoxide dismutase 1 levels and contributed to cellular death. These findings enhance our understanding of the intricate mechanisms underlying colitis and contribute to the development of novel therapeutic approaches for this challenging condition.

## Introduction

Inflammatory bowel disease (IBD) is a chronic inflammatory condition that significantly impacts individuals and presents with diarrhea, bloody stool, and unintended weight loss. This disorder comprises two primary types: ulcerative colitis (UC) and Crohn’s disease (CD)^1^. Although the affected population is steadily increasing, the exact etiology remains unclear, and environmental factors, immune system responses, and genetic predisposition are collectively considered risk factors for colitis^2,3^.

The pathogenesis of the disease involves an increase in intestinal macrophages^4,5^. These macrophages play a crucial role in the inflammatory response within the intestine by promoting the production of inflammatory cytokines, thereby contributing to the severity of the disease^6,7^. Reactive oxygen species (ROS) serve as one of the key activators of macrophages, playing a pivotal role in modulating their inflammatory response. Thus, increased macrophage activation and sustained ROS production can contribute to cell damage and prolonged inflammation, potentially exacerbating inflammatory disease conditions^8^. Therefore, understanding the interaction between macrophage activation and ROS generation is crucial in IBD.

HSPA9 (Mortalin, mtHsp70, PBP74, and Grp75) is a mitochondrial chaperone from the heat shock protein 70 family. HSPA9 is predominantly present in mitochondria and is essential in mitochondrial processes, including protein import, folding, and degradation. Additionally, it participates in the regulation of the oxidative stress response, mitochondrial membrane potential, energy generation, and intracellular transport^9,10^. A deficiency in HSPA9 has been associated with increased mitochondrial fission, disrupting the balance in mitochondrial dynamics and resulting in ROS elevations^11^.

Mitochondrial dysfunction in colitis has been explored in various research studies^12–14^, indicating that regulating mitochondrial fission can elevate colitis symptoms^15^. Additionally, ROS overproduction due to a disruption in mitochondria can induce oxidative stress, potentially causing cell damage and activating immune cells, including macrophages^16,17^. Building on the known function of HSPA9 in mitochondrial dynamics and the established connection between mitochondrial dysfunction and the progression of colitis, we investigated the impact of HSPA9 reduction on colitis.

Although the specific role of HSPA9 in inflammation has not been thoroughly investigated, its potential impact on mitochondrial dynamics and oxidative stress suggests a possible connection to colitis pathology. To investigate this, we studied *HSPA9*-downregulated mice (*HSPA9* heterozygous mice) in a dextran sodium sulfate (DSS)-induced colitis model, which is known to have similarities with human UC.

In our investigation, compared with the DSS-treated wild-type (WT) mice, DSS-treated *HSPA9* heterozygous (Het) mice demonstrated the aggravated symptoms of disrupted mucosal layer, increased immune cell infiltration, and decreased tight junction with increased cytokine levels. Furthermore, we observed an increase in macrophage infiltration and polarization in response to the STAT3 signal in Het mice.

Additionally, we observed that the reduction in HSPA9 influenced the regulation of superoxide dismutase 1 (SOD1), an enzyme that regulates free superoxide radicals. Histological analysis revealed that compared with the DSS-treated WT group, DSS-treated Het mice demonstrated lower SOD1 levels. Considering recent studies have reported the role of SOD1 in protecting the mucosal barrier and balancing the immune system in colitis^18^, the limited increase in SOD1 levels in Het mice, along with the other results, may support the contribution of *HSPA9* to the aggravation of colitis.

Taken together, our study provides insights into the impact of downregulated *HSPA9* in DSS-induced colitis, demonstrating its influence on macrophage activation, oxidative stress regulation, and mitochondrial fission. This new finding increases our understanding of the disease's mechanisms and may offer a novel strategy to manage the inflammation in colitis.

## Results

### Influence of *HSPA9* downregulation on the progression and severity of inflammatory bowel disease

The pivotal role of mitochondrial dysfunction in the initiation and progression of colitis has been reported^12–14^. Interestingly, *HSPA9* is reported to influence mitochondrial fission, a process crucial for maintaining mitochondrial homeostasis^11,19^. Moreover, the analysis of patient datasets revealed a significant association between reduced *HSPA9* expression and IBD.

**Figure 1:**
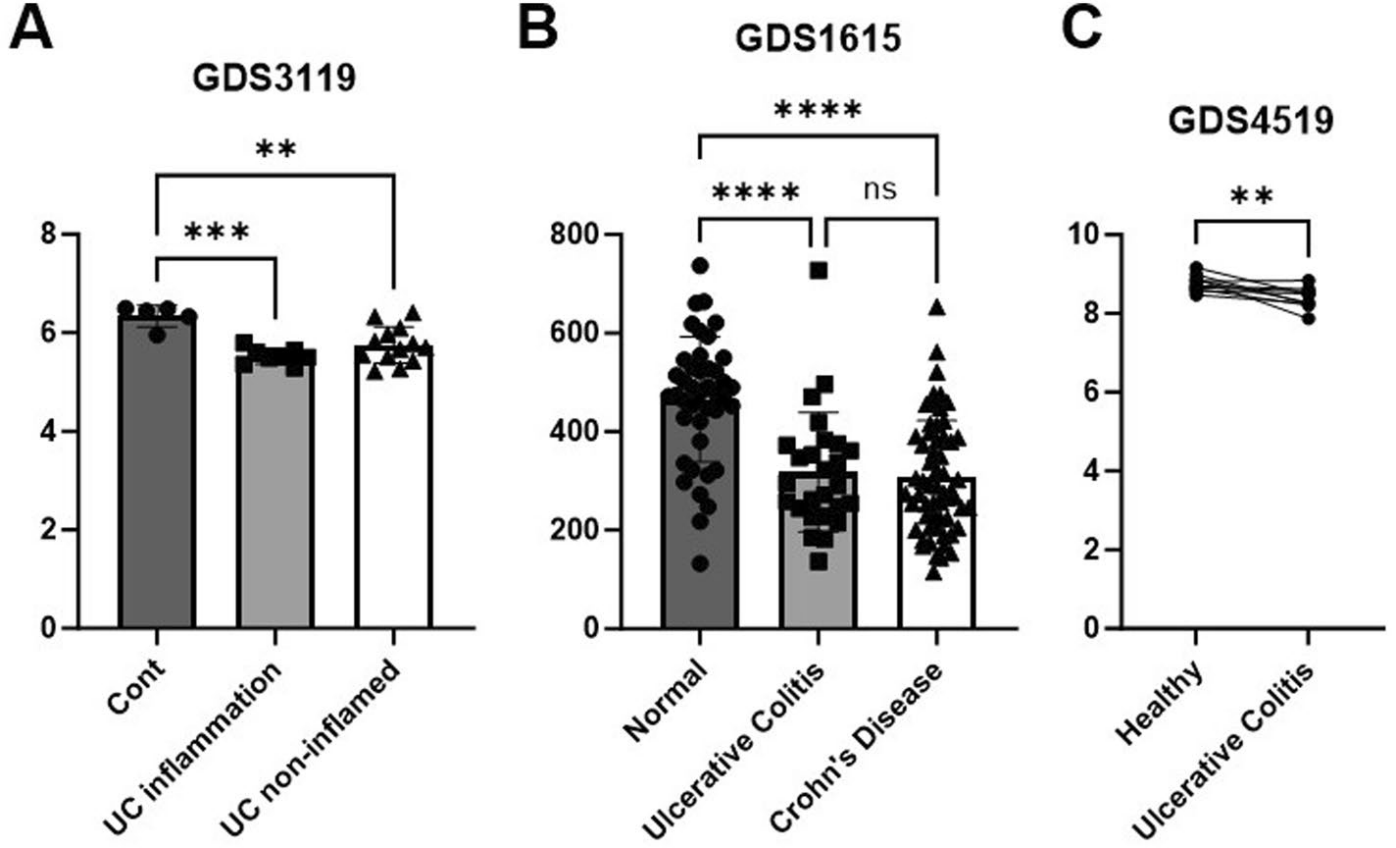
Diminished HSPA9 expression in inflammatory bowel Disease (IBD). (**A–C**) Comparative analysis of HSPA9 expression in human samples from patients with IBD using the Gene Expression Omnibus (GEO) on the National Center for Biotechnology Information (NCBI) website. (**A**) HSPA9 expression in colonic mucosa samples from patients with ulcerative colitis (UC) compared with controls from GEO datasets (GDS) 3119. (**B**) Examination of HSPA9 expression in peripheral blood mononuclear cells from patients with UC, patients with Crohn’s disease (CD), and healthy controls. (**C**) Comparison of HSPA9 expression in intestinal biopsies from twin pairs, where one individual is healthy and the other has UC. Data are presented as the mean ± standard deviation. Statistical significance was determined using an ordinary one-way ANOVA. *p < 0.01, **p < 0.005, and ***p < 0.0001.

Examination of the microarray data from the Gene Expression Omnibus (GEO) on National Center for Biotechnology Information (NCBI) revealed that, compared with healthy controls, patients with colitis demonstrated downregulation of HSPA9 expression. Specifically, the GSE3119 dataset indicated a decrease in HSPA9 expression in inflamed and noninflamed colonic mucosa with UC compared with their healthy counterparts (Fig. 1A).

Furthermore, the GSE1615 dataset that gathered information from the peripheral blood mononuclear cells of individuals diagnosed with both UC and CD, consistently supported the finding of decreased HSPA9 expression (Fig. 1B). Analysis of twins, where one sibling was diagnosed with UC while the other remained healthy, also revealed reduced HSPA9 levels in the intestinal biopsies of the twin affected by UC (Fig. 1C). Thus, we investigated the relationship between HSPA9 and colitis through a DSS-induced colitis mouse model.

We generated *HSPA9* knockdown mice using the CRISPR/Cas9 system. However, when Het mice were crossed to produce offspring, no homozygous mice were born. This outcome is consistent with reports in humans and mice where a > 50% knockdown of *HSPA9* is lethal^20–22^. However, the Het mice exhibit no significant growth retardation or defects, including their weight and size. Therefore, Het mice were used in this study.

**Figure 2:**
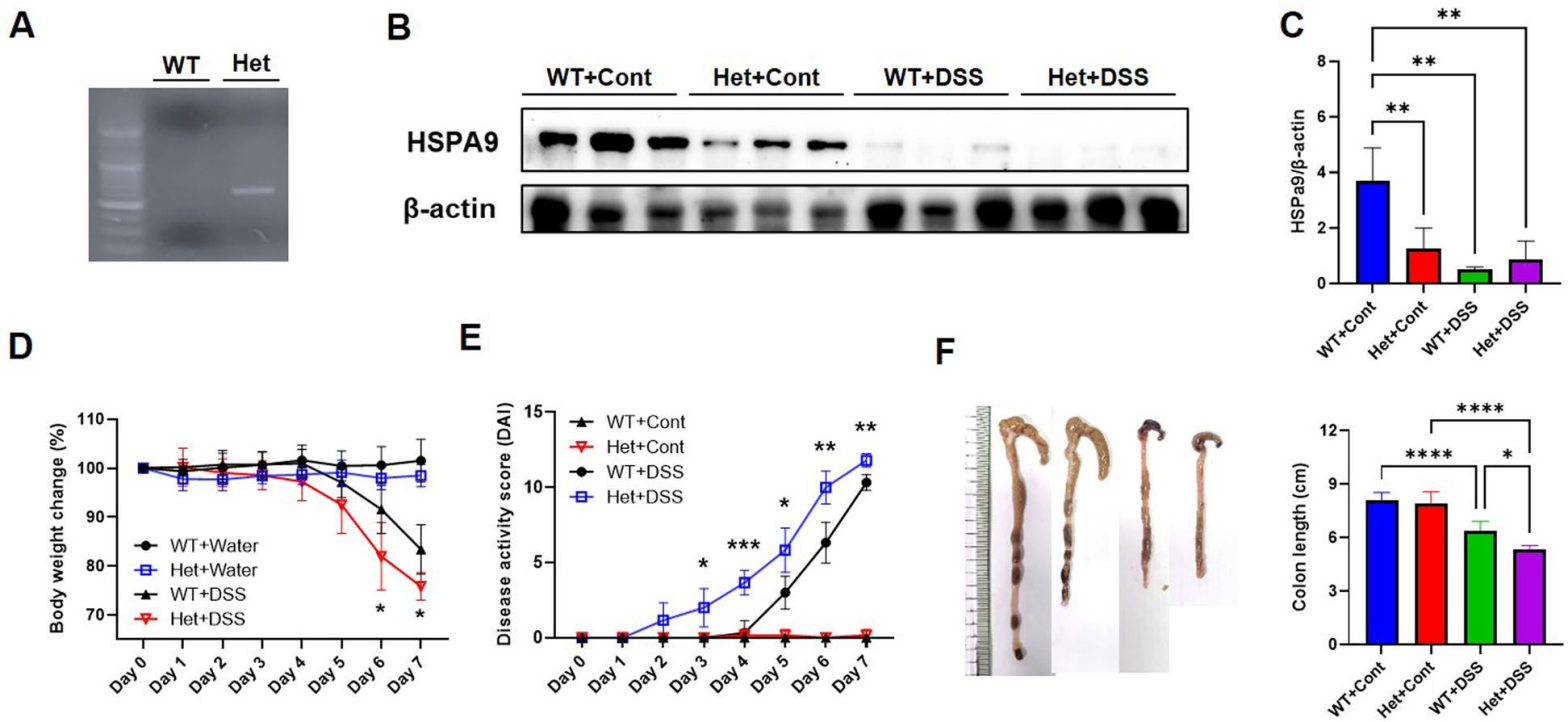
Impact of *HSPA9* downregulation on symptom severity in DSS-induced colitis. (**A**) Generation of *HSPA9*-downregulated mice using the CRISPR/CAS9 system. Verification of gene deletion in heterozygous (Het) mice was confirmed by PCR analysis. (**B**) Expression levels of *HSPA9* were presented by the western blot in colonic tissue samples from WT and Het mice, with or without DSS treatment. (**C**) Quantification of *HSPA9* expression from western blot, normalized to the expression of beta-actin. Statistical analysis was performed using a two-way ANOVA. (**D**) Body weight changes during DSS treatment from day 0 to day 7 were assessed. Statistical analysis was performed with a mixed-effects analysis. (**E**) DAI scores of the WT and Het mice, with or without DSS treatment from day 0 to day 7 were presented. Statistical analysis was performed with mixed-effects analysis. (**F**) Photographs and length analysis of the colon were presented to evaluate colon shortening related to colitis severity in WT and Het mice. Statistical analyses was performed with a two-way ANOVA. *p < 0.05 and **p < 0.01.

For the analysis of the mice genotypes, primers designed to detect Het mice were used for PCR analysis (Fig. 2A). To investigate *HSPA9* deficiency in colitis, we employed a DSS-induced mouse model, which is a widely used chemical for experimental colitis mouse models. Administering DSS to mice initiates epithelial degradation and produces erosive lesions in the intestinal mucosa, resulting in the weakening of the epithelial barrier, loss of tight junction components, such as junctional complex (ZO-1), increased immune cell infiltration, and the release of inflammatory cytokines (IL6, TNFα, IFNγ, IL1β, and IL12). Collectively, the DSS-induced colitis model mice presented with symptoms that included weight loss, diarrhea, and bloody stool.

In our study, both WT and Het mice were administered either water or DSS for seven days. After the treatment period, colon tissue samples were collected from each group for further analysis. In the control group, the expression levels of *HSPA9* in colon tissue were significantly lower in the Het mice compared with WT mice (Fig. 2B, C). A significant decrease in *HSPA9* expression was observed in the DSS-treated WT mice when compared with the control WT mice.

In the body weight alteration measurement from days 0–7, there was no significant difference between WT and Het mice that were administered water. However, DSS-induced Het mice exhibited an early 5% weight loss compared with DSS-induced WT mice, and this trend intensified on days 6 and 7, exhibiting statistical significance (Fig. 2D). Moreover, DSS-induced Het mice demonstrated a significant and earlier increase in disease activity index (DAI) score, showing a higher elevation from day 3 to day 7 compared with DSS-induced WT mice (Fig. 2E). On day 8, DSS-induced Het mice displayed a shorter colon than the DSS-induced control mice (Fig. 2F).

### *HSPA9* downregulation in DSS-induced colitis exacerbates the histological phenotypes

**Figure 3:**
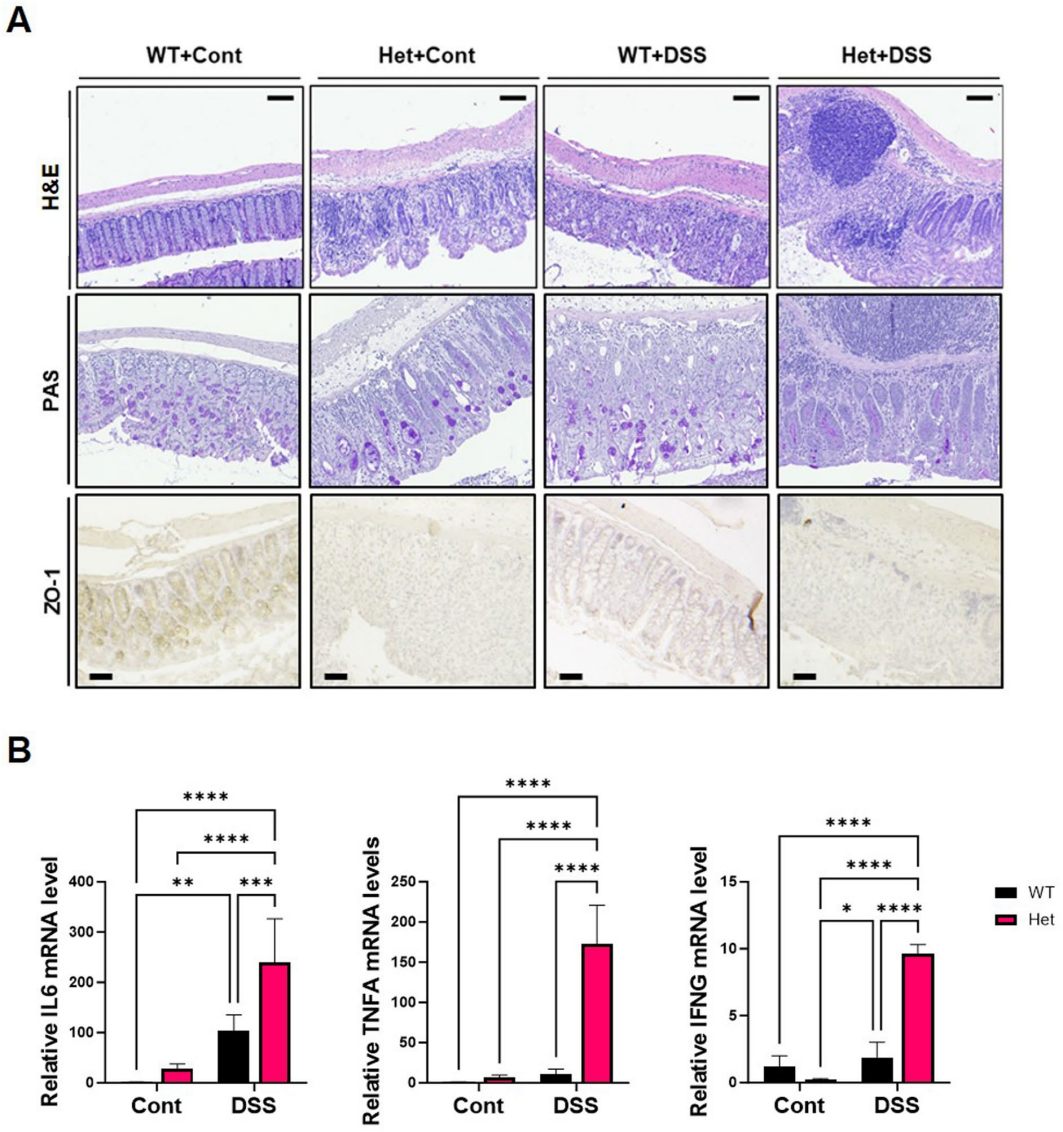
*HSPA9* downregulation aggravates colonic histopathology and increases cytokine production in DSS-induced colitis. (**A**) To elucidate the impact of *HSPA9* downregulation in DSS-induced colitis, we performed histological evaluation of colon tissue. Hematoxylin & eosin (H&E) staining shows structural integrity and immune cell infiltration while Periodic acid–Schiff (PAS) staining revealed the status of mucin-secreting goblet cells. The expression level of ZO-1, a tight junction marker, was visualized through immunohistochemistry. (**B**) Inflammatory cytokine levels including *IL6*, *TNFA*, *and IFNG,* in colonic tissue were evaluated using qRT-PCR. Data are presented as the mean ± standard deviation. Statistical analysis was performed with a two-way ANOVA *p < 0.05, **p < 0.01, ***p < 0.005, and ****p < 0.0001.

To investigate the progression of IBD in relation to *HSPA9*, histological analysis was conducted using DSS-induced Het mice. Hematoxylin and eosin (H&E) staining revealed that DSS treatment induced colitis in the WT and Het mice, characterized by intestinal epithelial damage, goblet cell depletion, and inflammatory cell infiltration (Fig. 3A). In the DSS-treated groups, Het mice exhibited more severe immune cell infiltration than WT mice. Interestingly, even without DSS treatment, Het mice displayed some damage in crypts and increased immune cell infiltration.

Periodic acid–Schiff (PAS) staining also confirmed increased symptomatic severity in Het mice, including disruption of the mucus layer and a reduced number of goblet cells, both with and without DSS treatment (Fig. 3A). Additionally, compared with WT mice, Het mice demonstrated reduced levels of the tight junction protein zonula occludens-1 (ZO-1). Thus, it suggests an increase in intestinal permeability and immune cell infiltration^23,24^.

Given the observed increase in immune cell infiltration in the control and DSS-treated Het mice groups compared to matched WT mice, we investigated the cytokine levels using quantitative RT-PCR. The results revealed a dramatic increase in *IL6*, *TNFA*, and *IFNG* in the DSS-treated Het mice compared with DSS-treated WT (Fig. 3B). These results indicate that the downregulation of *HSPA9* plays a crucial role in modulating the severity of colitis symptoms, influencing the immune response, mucosal integrity, and cytokine expression.

### Exacerbated macrophage infiltration and STAT3 activation in reduced *HSPA9* mouse model

To identify specific cell types infiltrating the colon tissue, we performed immunofluorescence staining. Given that the dataset analysis shows a significantly diminished expression level of *HSPA9* in the peripheral blood mononuclear cells, we performed immunofluorescence staining to examine the macrophages.

**Figure 4:**
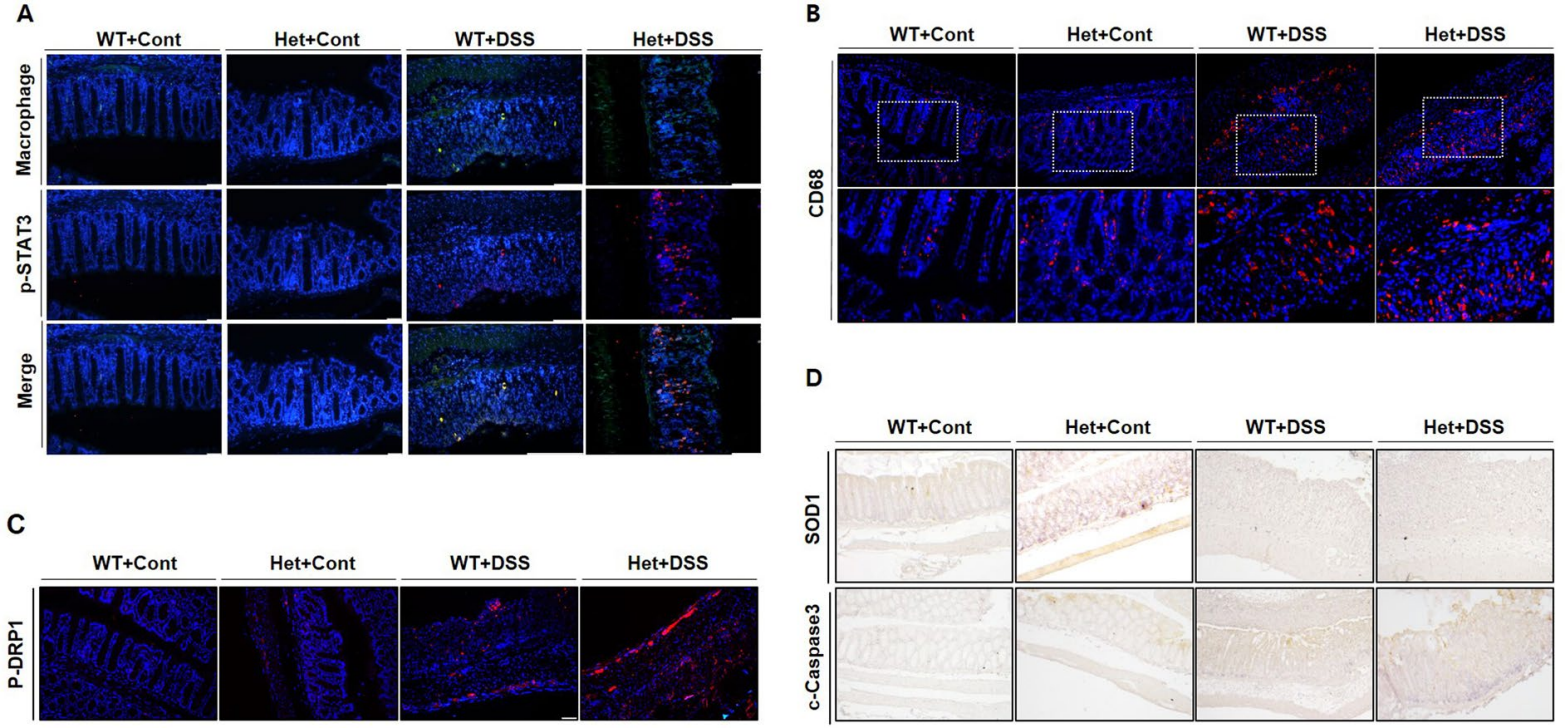
*HSPA9* downregulation increases macrophage polarization and cell death in DSS-induced inflammatory colitis. (**A**) Immunofluorescence staining was conducted to demonstrate the expression levels of macrophages (Green) and p-STAT3 (Red). (**B**) CD68 expression in the colon tissue was evaluated using immunofluorescence staining (Red). (**C**) Mitochondrial fission was assessed by p-DRP1 (s616) staining (Red) through immunofluorescence analysis. (**D**) Immunohistochemical staining demonstrated a decrease in SOD1 and increased cleaved caspase-3 levels in DSS-treated Het mice, indicating alterations in programmed cell death processes within the colon tissue. The blue fluorescence in the images corresponds to DAPI staining of the nuclei.

The result revealed an elevated number of macrophages in the Het mice groups compared with the WT mice groups, both with and without DSS treatment (Fig. 4A). Moreover, we investigated macrophage activation. Phosphorylation of STAT3 plays a pivotal role in macrophage polarization. In the context of cancer, increased p-STAT3 levels are associated with M2 polarization. However, in situations involving microorganisms, elevated p-STAT3 influences macrophage M1 polarization^25^. Furthermore, in microglia, which represents another form of macrophage, p-STAT3 influences the increase in mitochondrial fission and cytokine production^26–28^.

To investigate macrophage activation, we stained both macrophages and the p-STAT3 marker. An increased number of macrophage cells was found in Het mice compared with WT mice under both control and DSS conditions. Moreover, phosphorylation of STAT3 was significantly upregulated in the macrophages of Het mice after the DSS treatment. Taken together, the results suggest that the invasion and activation of macrophages can be upregulated by *HSPA9* reduction.

CD68, a transmembrane glycoprotein that is predominantly expressed on the surface of macrophages, serves as a crucial clinical marker of maturation. In patients with IBD, disease severity is significantly associated with macrophages, and a high correlation was demonstrated with the macrophage marker CD68^29^. This implies that reduced CD68-positive macrophages contribute to the therapeutic response and healing process, whereas increased CD68-positive macrophages play an important role in IBD severity. Thus, we explored the CD68 expression in DSS-induced colitis model mice.

Immunofluorescence staining results showed an increased expression in Het mice compared with WT mice under control conditions (Fig. 4B). In the DSS-treated groups, Het mice displayed a significant increase in CD68-positive cells compared with their WT counterparts. This increment, along with the earlier results, suggests that *HSPA9* downregulation possibly exacerbates colitis by enhancing infiltration and triggering macrophage activation via the STAT3 pathway. This observation is consistent with the increased cytokine levels measured earlier, indicating heightened inflammation (Fig. 3B).

### Upregulated mitochondrial fission and apoptosis induction upon *HSPA9* reduction in DSS-induced colitis

Dynamin-related protein 1 (DRP1) is a pivotal regulator of mitochondrial dynamics, playing a central role in mitochondrial fission and fusion processes^30,31^. The phosphorylation of p-DRP1 (S616) promotes mitochondrial fission, influencing the number of mitochondria. The regulation of p-DRP1 (S616) can significantly impact cellular energy production and overall cell survival.

Under control conditions, Het mice exhibited increased levels of p-DRP1 (S616) compared with WT mice (Fig. 4C). This elevation became even more pronounced in the Het mice of the DSS-treated group, implying a heightened impact of mitochondrial fission. This observation suggests that the increased burden on mitochondrial dynamics may have contributed to the severity of symptoms observed in Figs. 2 and 3.

Interestingly, several studies have demonstrated the interaction between DRP1 and SOD1 levels. DRP1 can influence SOD1 levels, and conversely, SOD1 levels can affect DRP1^32–34^. SOD1 is an essential enzyme that protects cells from oxidative stress by breaking down superoxide radicals into less harmful molecules, such as oxygen and hydrogen peroxide^35^. Dysregulation of SOD1 can lead to the accumulation of superoxide radicals, resulting in damage to crucial cellular components, including proteins, lipids, and DNA, through a process known as oxidative stress^36^.

The influence of SOD1 on colitis has been attributed to its capacity to suppress oxidative stress, immune response, and cell death^18^. Consequently, a low expression of SOD1 may contribute to the severity and progression of colitis. In the histological analysis, DSS treatment increased SOD1 levels in the WT mice. However, the increased SOD1 levels in the Het mice were considerably lower than those of the WT mice (Fig. 4D). Moreover, an increase in cleaved caspase-3, a crucial protein involved in programmed cell death, was also observed in DSS-treated Het mice relative to DSS-treated WT mice (Fig. 4D)^37,38^.

The increase in p-DRP1 (S616) and decrease in SOD1 due to an *HSPA9* reduction may have synergistically contributed to elevating cleaved caspase-3, indicating an escalation of cell damage and death. These findings suggest that the reduction of *HSPA9* disrupts mitochondrial homeostasis and impairs oxidative stress regulation, leading to increased superoxide radicals and apoptosis, thereby contributing to the progression of colitis.

## Discussion

IBD is influenced by multiple factors, including genetic predispositions, environmental factors, and immune system dysregulation. Although its exact etiology remains elusive, the substantial increase in intestinal macrophages during the disease progression has been widely recognized^5,7,39–41^. Activated macrophages, which can be stimulated by ROS, contribute significantly to the inflammatory process and the severity of the disease^4–6,39^.

HSPA9, a mitochondrial chaperone of the heat shock protein 70 family, is a key regulator of mitochondrial processes, including protein import, folding, and degradation. Additionally, it plays a pivotal role in responding to oxidative stress and peroxisomal autophagy^42,43^. Previous research has suggested that *HSPA9* deficiency can disrupt mitochondrial homeostasis, promote ROS production and oxidative stress, and activate immune cells, including macrophages^9,19,44^. Moreover, we observed a consistent pattern of downregulated HSPA9 expression in datasets of patients with colitis (Fig. 1). This finding emphasizes the possible involvement of HSPA9 in the progression of the disease.

Based on this observation, we investigated the connection between HSPA9 and colitis using an *HSPA9* knockdown mouse model. In DSS-induced colitis, reduced *HSPA9* expression was observed in WT mice, suggesting a relationship between *HSPA9* and disease progression (Fig. 2). Compared with WT mice, Het mice displayed more severe symptoms after the DSS treatment, including weight loss and reduced colon length. Additionally, *HSPA9* downregulation had a significant impact on pathology and cytokine levels (Fig. 3).

Moreover, compared with the DSS-treated WT mice, DSS-treated Het mice demonstrated increased immune cell infiltration, macrophage polarization, mitochondrial fission, and cell death markers. This result supports the notion that *HSPA9* is critical in modulating immune responses, maintaining mucosal integrity, and affecting colitis symptoms via mitochondrial dysfunction.

Macrophages play an integral role in maintaining the stability of the intestinal environment and regulating immune responses, particularly in the context of colitis^4–6,39–41,45^. In ideal circumstances, immune responses are expected to facilitate the resolution of inflammation and expedite tissue recovery. However, in colitis, prolonged immune system activation disrupts intestinal homeostasis.

In this study, we revealed the connection between *HSPA9* downregulation and macrophage activation. In inflammation involving microorganisms, heightened p-STAT3 levels impact macrophage M1 polarization^25^. Additionally, it is reported that increased p-STAT3 levels in the microglia, resident macrophages of the central nervous system, promote mitochondrial dysfunction and cytokine production^26–28^. Macrophages that polarized toward M1 contribute to intestinal damage and homeostasis imbalance^46,47^. Colon tissue from Het mice showed increased M1 polarization of macrophages through the STAT3 pathway (Fig. 4A, B). Taken together, these results indicate that *HSPA9* reduction further exacerbates colitis, leading to a more severe phenotype of DSS-induced colitis by influencing the macrophages activation.

*HSPA9* downregulation influences mitochondrial homeostasis by altering DRP1 phosphorylation (S616) in DSS-induced colitis (Fig. 4). This alteration in the mitochondrial dynamics, characterized by increased fission, may lead to an elevated production of mitochondrial ROS, thereby contributing to the manifestation of severe symptoms in the DSS-induced colitis model.

Interestingly, Het mice, even when only water was administered, exhibit mild colitis features compared with the WT mice in the histological analysis. This included increased immune cell infiltration, disrupted colonic mucosa, and reduced expression of tight junction markers. Additionally, a relatively higher level of proinflammatory cytokines, such as *IL6* and *TNFA*, was demonstrated in the Het mice compared with the WT mice in the control groups. This suggests that, even in the absence of an inducer, *HSPA9* reduction may impact mitochondrial function and provoke an immune response, potentially inducing tissue damage. While this study suggests this possibility, it has not conclusively demonstrated this effect. Extending the experimental period for *HSPA9* Het mice may help elucidate the suggested possibility more clearly. We intend to investigate these aspects in aged Het mice in further studies.

In addition, we discovered a novel aspect of *HSPA9*, namely its relation to SOD1, one of the key enzymes involved in mitigating oxidative stress^18,36^. The concept of investigating SOD1 originated from GDS3119, a dataset involving colonic mucosa samples from patients with UC. Given that inflammation in UC primarily occurs in the colonic mucosa^48^, we investigated the factors related to *HSPA9* and immune responses. Interestingly, we found a significant correlation between the expression of *HSPA9* with SOD1, which was greater than with SOD2, in GDS3119. Thus, we investigated the SOD1 level in Het mice.

In the colonic samples of DSS-treated mice, Het mice did not demonstrate a significant increase in SOD1 levels compared to WT mice, indicating that the reduction in *HSPA9* influenced the regulation of SOD1 under DSS treatment (Fig. 4). It is unclear whether the decrease in SOD1 levels is directly related to HSPA9 levels or other factors. However, considering that SOD1 is widely distributed in the cell, including the mitochondrial intermembrane space^36^, and there are several reports indicating the interaction between DRP1 and SOD1, this suggests the possibility that the reduced HSPA9 levels increased mitochondrial fission, thereby influencing SOD1 levels that lead to an aggravated inflammatory response in colitis.

Further investigation is required to clarify the mechanism between *HSPA9* and SOD1 expression. Nevertheless, the connection between *HSPA9* and the regulation of oxidative stress through SOD1 enhances our understanding of the role of *HSPA9* in regulating mitochondrial ROS and colitis symptoms.

In conclusion, our research demonstrated the critical role of *HSPA9* in the development and progression of colitis. An enhanced understanding of the relationships among *HSPA9* expression, mitochondrial dynamics, macrophage activation, and ROS regulation in colitis provide valuable insights that may facilitate innovative therapeutic strategies. These findings emphasize the potential for targeted approaches to alleviate the severity of IBD symptoms and improve patient outcomes.

## Methods

### Data set analysis

The expression of *HSPA9* was investigated using microarray experiment data obtained from the GEO on the NCBI website (https://www.ncbi.nlm.nih.gov/geo/). The analysis encompasses datasets for various conditions: GDS3119 (colonic mucosa samples from 21 patients with UC and 5 controls), GDS1615 (peripheral blood mononuclear cells from 27 patients with UC, 59 with CD, and 42 controls), and GDS4519 (intestinal biopsies from 13 twin pairs, with one individual being healthy and the other with UC).

### Mice

*HSPA9* knockdown mice were generated using the CRISPR/Cas9 system with the genetic background of C57BL/6 mice, targeting exon 2–4 deletion. The genotype was confirmed by PCR targeting the WT and Het alleles using genomic DNA. PCR amplification was performed with *HSPA9* primers (F: 5′-CAC ACA TGG GGT TTT GTG TGC-3′, R: 5′-CTG GGA AGG CAA AAA TGG GCT-3′). Mice aged 10–12 weeks were housed under standard laboratory conditions (22 °C ± 25 °C, 12 h/12 h light/dark cycle, 50%–60% humidity). Because of the lethality associated with complete *HSPA9* knockout, experiments were conducted using Het mice. All animal experiments adhered to the guidelines for animal experimentation of the Kyungpook National University Animal Care and Use Committee and received approval from the ethics committee (Daegu, Korea). All experiments were performed in a manner consistent with the ARRIVE guidelines (https://arriveguidelines.org).

### Induction of acute colitis with DSS

DSS is widely used in experimental colitis mouse models because of its reproducibility and its similarity with human UC^49,50^. Administering DSS induces epithelial degradation and erosive lesions in the intestinal mucosa, thereby weakening the epithelial barrier with the increased permeability^51^. This leads to the heightened colonization of microorganism, the loss of tight junctions, and the increased infiltration of immune cells with the release of inflammatory cytokines^52^. Mice subjected to DSS display symptomatic features, such as weight loss, diarrhea, and bloody stools.

To induce colitis, 10–12-week-old WT and Het mice were administered 2.5% DSS (MW 36,000–50,000, MP Biomedicals, CA, USA) in their drinking water for seven days to induce acute colitis. Throughout the administration period, their weight and stool conditions were regularly observed and scored using the DAI. On day 8, the mice were euthanized via intraperitoneal administration of avertin (300 mg/kg), and tissue samples were harvested for further analysis.

### Disease activity index

Daily symptoms, such as weight loss, stool consistency (normal, loose, or diarrhea), and blood in the stool, were monitored to evaluate the progression of colitis. As described by Murthy et al.^53^, body weight loss was assessed using the following scoring system: 0, no weight loss compared with the initial weight; 1, 1%–5% weight loss; 2, 5%–10% weight loss; 3, 10%–20% weight loss; and 4, > 20% weight loss. Stool consistency was categorized as follows: 0, normal (solid pellet); 1, soft but pellet-shaped; 2, loose stool with some solidity; 3, loose stool with signs of liquid consistency; and 4, watery diarrhea. Rectal bleeding was scored as follows: 0, no sign of blood; score 1, no bleeding; 2, slight bleeding; 3, bloody diarrhea; and 4, gross bleeding. The presence of blood in the stool was examined visually and confirmed through a luminal reaction test^53,54^. DAI scores were derived by combining the scores for body weight loss, stool consistency, and gross bleeding.

### Western blot analysis

Colon tissues were extracted using the Pro-Prep lysis buffer according to the manufacturer’s instructions (iNtRON Biotechnology, Seoul, Korea). Samples were separated by 10–12% SDS-PAGE, and the proteins were transferred to a PVDF membrane. The blots were cut prior to hybridization with antibodies during the blotting. The membranes were then incubated with the following primary antibodies: *HSPA9* (818801, BioLegend, CA, USA) and β-actin (sc-47778, Santa Cruz Technology, CA, USA). Subsequently, the membranes were incubated with the corresponding secondary antibodies. Chemiluminescent signals were developed using an ECL detection kit.

### Histology analysis

Histological analysis was conducted to assess DSS-induced histological damage in the model mice. Briefly, distal colon tissues were fixed in 4% paraformaldehyde and embedded in paraffin. The paraffin blocks were then cut into 8-µm slices and stained with H&E for initial evaluation. Further investigation was conducted through immunohistochemistry (IHC). For IHC staining, deparaffinized and rehydrated sections were treated with a 10 mM citrate buffer (pH 6.0) for antigen retrieval. The sections were incubated overnight at 4 °C with primary antibodies, including ZO1 (sc-8146, Santa Cruz Technology), macrophage (sc-101447, Santa Cruz Technology), CD68 (#97,778, Cell Signaling Technology), p-DRP1 (S616) (#611738, BD), SOD1 (sc-11407, Santa Cruz Technology), cleaved caspase-3 (#9661S, Cell Signaling Technology), and p-STAT3 (#9131, Cell Signaling Technology). Subsequently, the slides were incubated with the corresponding secondary antibodies.

### Quantitative RT‑PCR

Quantitative RT-PCR (qRT-PCR) was conducted to assess the expression of proinflammatory cytokines, including *IL6* (F: 5’-GAG GAT ACC ACT CCC AAC AGA CC-3’, R: 5’- AAG TGC ATC ATC GTT CAT ACA-3’), *TNFA* (F: 5’-TGT GCT CAG AGC TTT CAA C-3’, R: 5’-GCC CAT TTG AGTCCT TGA TG-3’), and *INFG* (F: 5’-CAA CAG CAAGGC GAA AAA GG-3’, R: 5’-TGG TGG ACC ACT CGG ATG-3’). Total RNA was isolated from distal colon specimens, homogenized in TRI-Solution (TS100-001, BSK-BIO Technology, Daegu, Korea), and extracted following the manufacturer’s instructions. Reverse transcription was performed using the PrimeScript™ 1st Strand cDNA Synthesis Kit (6110A, TaKaRa Bio Inc., Shiga, Japan). qRT-PCR was conducted using SYBR Premix Ex Taq (RR420A, TaKaRa Bio Inc., Shiga, Japan) and analyzed on a StepOnePlus™ Real-Time PCR System (Applied Biosystems, Foster City, CA). The relative mRNA expression levels were calculated using the 2^−∆∆Ct^ method and normalized to β-actin.

### Statistical analysis

All graphs and statistical analyses were performed using GraphPad Prism version 9.1.2 (GraphPad Software, San Diego, CA, USA). The GEO datasets were subjected to comparison using one-way ANOVA and Tukey’s multiple comparison test. The data collected from day 0 to 7 were compared using mixed-effects analysis and Tukey’s multiple comparison test. For the comparison of WT and Het mice treated with water or DSS, two-way ANOVA and Tukey’s multiple comparison test were used. Results are presented as the mean ± standard deviation. Statistically significant distinctions among the experimental groups were identified using the Student’s *t*-test, with *p < 0.05, **p < 0.01, ***p < 0.005, and ****p < 0.0001.

### Supplementary Information

Supplementary Information.

## Data Availability

The datasets (Accession Numbers: GSE3119, GSE1615, GDS4519) analyzed during the current study are available in the Gene Expression Omnibus (GEO) [Internet]. Bethesda (MD): National Center for Biotechnology Information (US); 1999—[cited 2023 09 27]. Available from: https://www.ncbi.nlm.nih.gov/geo/
