# Supplementary figures and images for: HSPA9 reduction exacerbates symptoms and cell death in DSS-Induced inflammatory colitis
Source: Sci Rep. 2024 Mar 11;14:5908. doi: 10.1038/s41598-024-56216-w (PMC10928168; doi:10.1038/s41598-024-56216-w)

## Set1

Hspa9

Marker

70kda

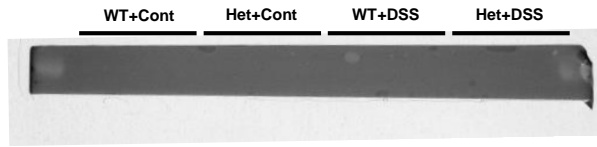

72kda →

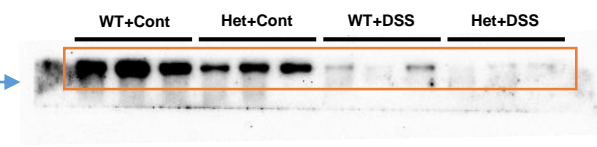

$\beta$ -actin

Marker

55kda

40kda

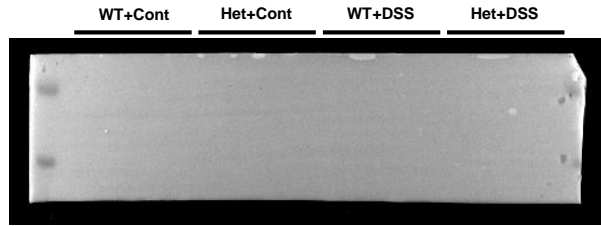

43kda →

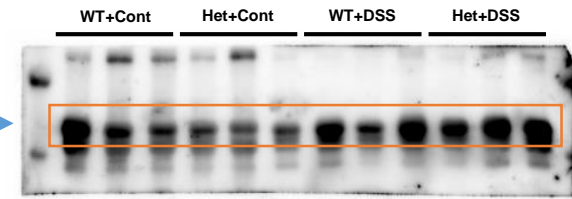

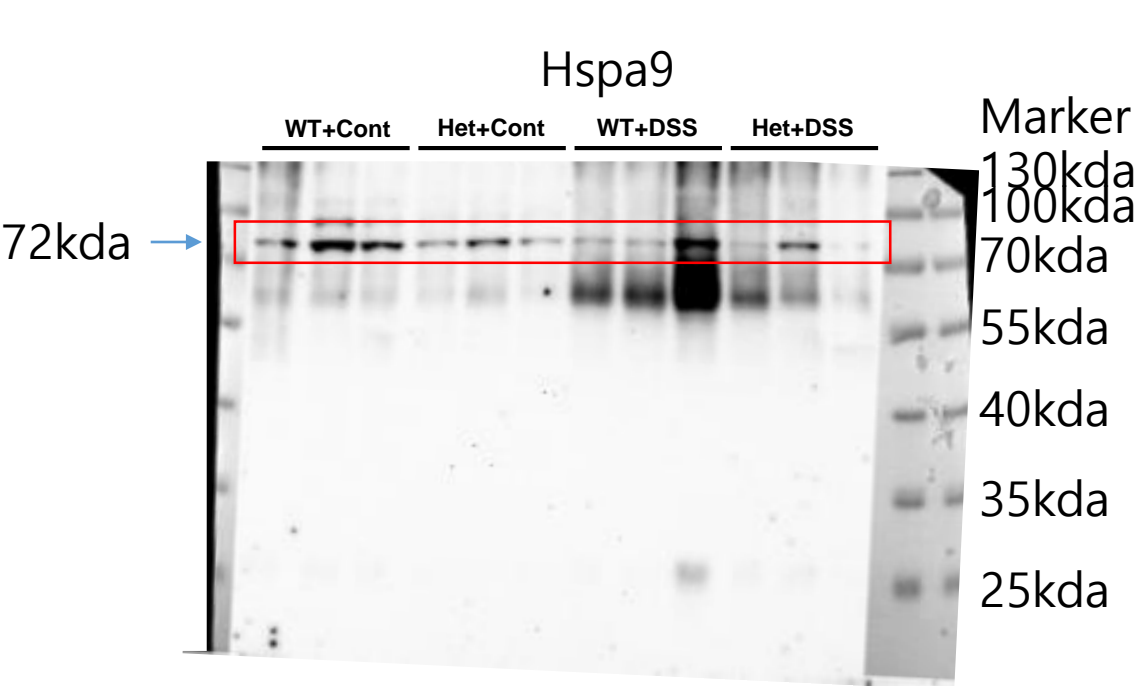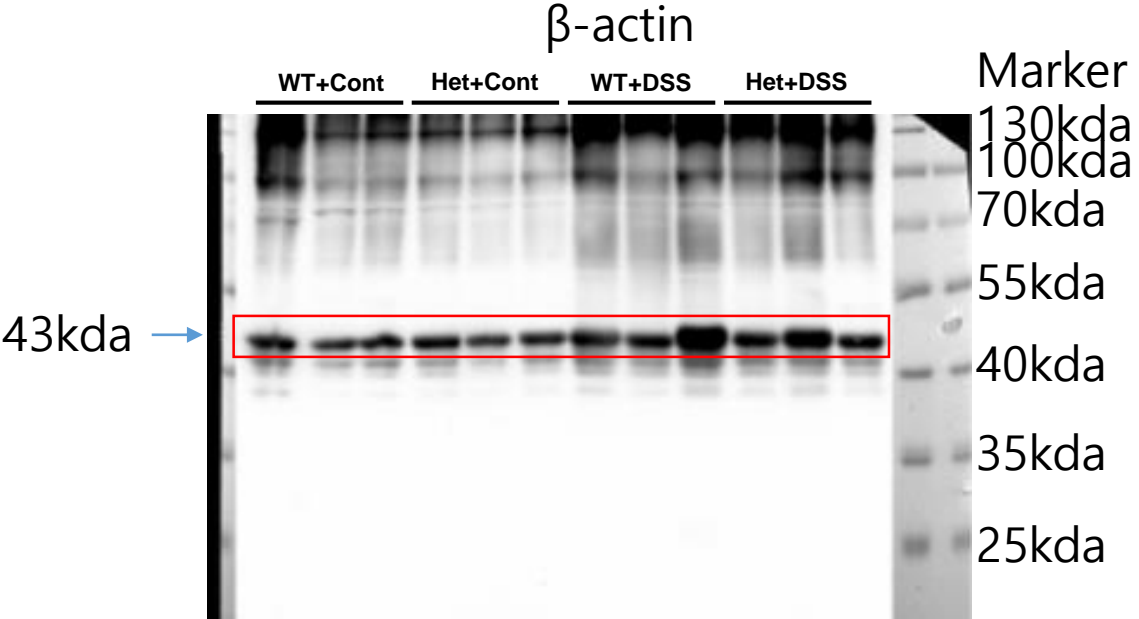

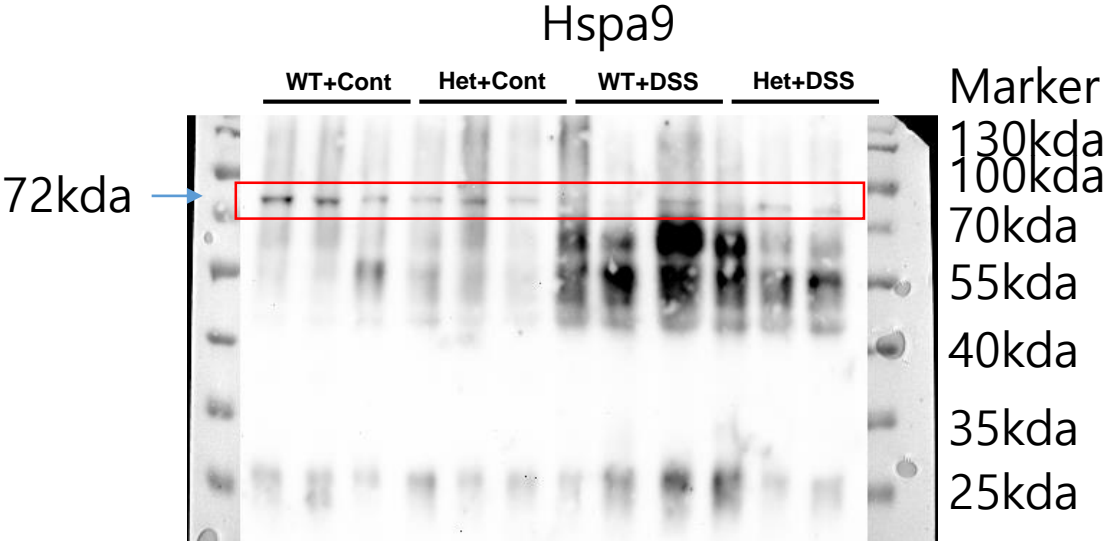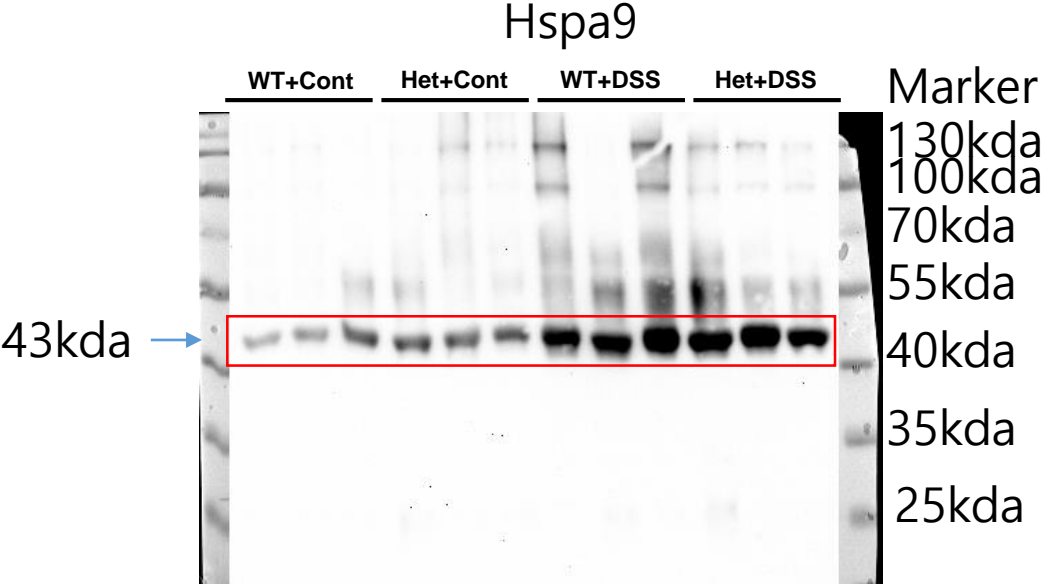

Supplement: Supplementary file 1 — Supplementary Information. [file 41598_2024_56216_MOESM1_ESM.pdf]
